# spKAS-seq reveals R-loop dynamics using low-input materials by detecting single-stranded DNA with strand specificity

**DOI:** 10.1126/sciadv.abq2166

**Published:** 2022-11-30

**Authors:** Tong Wu, Ruitu Lyu, Chuan He

**Affiliations:** ^1^ Department of Chemistry, University of Chicago, Chicago, IL, USA.; ^2^ Howard Hughes Medical Institute, University of Chicago, Chicago, IL, USA.; ^3^ Department of Biochemistry and Molecular Biology, Institute for Biophysical Dynamics, University of Chicago, Chicago, IL, USA.

## Abstract

R-loops affect transcription and genome stability. Dysregulation of R-loops is related to human diseases. Genome-wide R-loop mapping typically uses the S9.6 antibody or inactive ribonuclease H, both requiring a large number of cells with varying results observed depending on the approach applied. Here, we present strand-specific kethoxal-assisted single-stranded DNA (ssDNA) sequencing (spKAS-seq) to map R-loops by taking advantage of the presence of a ssDNA in the triplex structure. We show that spKAS-seq detects R-loops and their dynamics at coding sequences, enhancers, and other intergenic regions with as few as 50,000 cells. A joint analysis of R-loops and chromatin-bound RNA binding proteins (RBPs) suggested that R-loops can be RBP binding hotspots on the chromatin.

## Teaser

spKAS-seq was developed to map genome-wide R-loops by detecting single-stranded DNA with strand specificity.

## INTRODUCTION

R-loop bears a unique triple-stranded nucleic acid structure, forming by a single-stranded RNA invading a DNA duplex and annealing with the cDNA strand. Aberrant R-loop formation has been linked to human diseases (*1*, *2*). For instance, R-loops at trinucleotide repeats were shown to associate with fragile X syndromes (*3*–*5*). Mutations in human *BRCA1* and *BRCA2* genes could increase R-loop levels throughout the genome, which induces DNA damage and increases risks for cancer (*6*–*8*). The molecular mechanisms behind R-loop functions are complex and can be context dependent (*9*). R-loops at promoters could activate or repress transcription by protecting DNA from methylation (*10*, *11*) or by altering the DNA binding affinity of transcription factors (TFs) and chromatin remodelers (*12*–*14*). R-loops at the 3′ end of genes were reported to facilitate transcription termination by stalling RNA polymerase II (Pol II) or inducing repressive histone marks at termination sites (*15*–*17*). Certain R-loops at intergenic regions could induce DNA replication stress and affect DNA damage responses (*18*–*22*).

Precise mapping of R-loops is critical to understanding R-loop functions. Genome-wide R-loop detection primarily relies on RNA-DNA duplex enrichment using the S9.6 monoclonal antibody (*11*, *13*, *23*–*28*) or catalytically inactive ribonuclease H (RNase H) (*29*–*31*), followed by high-throughput sequencing. These methods have been effective in revealing R-loop functions; however, immunoprecipitation-based approaches usually require millions of cells and may not be able to study biological processes with limited input materials. In addition, the S9.6 antibody and inactive RNase H appear to exhibit preferences for different R-loop sequences with varying results observed between different methods (*9*). For instance, in DNA:RNA immunoprecipitation sequencing (DRIP-seq), R-loop signals were detected across the entire gene-coding regions. Whereas, bis–DRIP-seq (*26*) and R-ChIP (*29*) detect R-loops almost exclusively at promoter regions. Therefore, it is highly desirable to have an R-loop mapping method that does not rely on S9.6 antibody and RNase H and can work in live cells using low-input materials.

We have recently developed kethoxal-assisted single-stranded DNA (ssDNA) sequencing (KAS-seq) for genome-wide mapping of ssDNA using as few as 1000 cells (*32*). N_3_-kethoxal labels the N1 and N2 positions of guanines and, thus, only reacts with ssDNA but not double-stranded DNA (dsDNA) (*33*). In transcription bubbles, N_3_-kethoxal reacts with both strands of DNA when nascent RNA is not base pairing with the template strand of DNA; however, in R-loop, it only labels the exposed strand of DNA but not the other strand that forms the RNA-DNA duplex. We reasoned that, when combining KAS-seq with strand-specific enrichment and library construction, we can detect asymmetric DNA strand exposure as a signature for R-loop identification. Because kethoxal can label ssDNA in live cells, this approach could detect R-loops in vivo without the need for cell lysis or permeabilization.

Here, we present strand-specific KAS-seq (spKAS-seq) that enables R-loop mapping with a robust covalent chemistry using 50,000 cells. spKAS-seq detects strong R-loops around transcription start sites (TSSs) as well as signals at gene bodies and the 3′ end of coding regions. spKAS-seq also identifies R-loops at enhancers and other intergenic regions and reveals temporally resolved R-loop dynamics in response to transcription perturbations. We also found that a portion of chromatin-binding RNA binding proteins (RBPs) show high chromatin immunoprecipitation sequencing (ChIP-seq) peak density on R-loop regions, suggesting a potential connection between R-loop and RBP binding.

## RESULTS

### spKAS-seq maps ssDNA with strand specificity

**Fig. 1.**
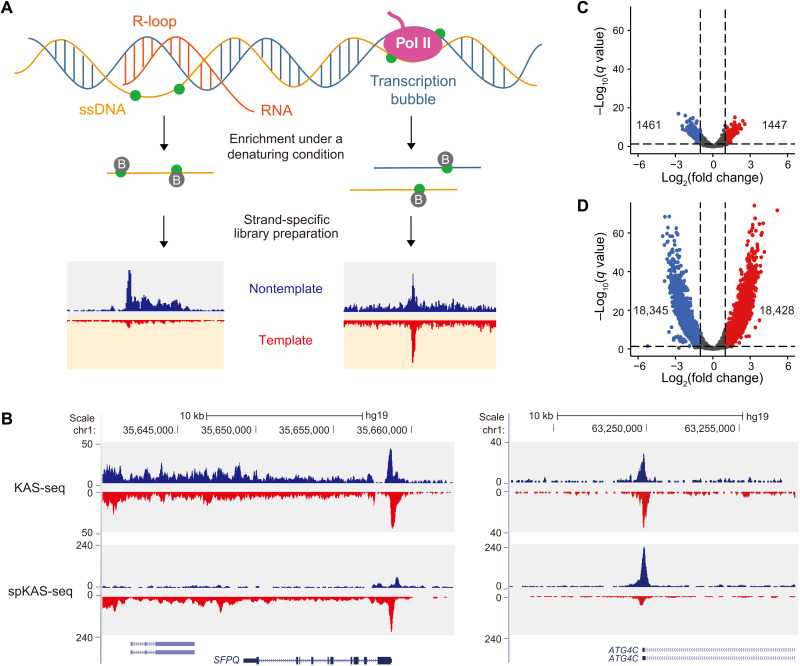
spKAS-seq for strand-specific ssDNA mapping and R-loop detection. (**A**) A schematic illustration of the principle that spKAS-seq distinguishes R-loops from transcription bubbles. The RNA-DNA hybrid duplex in R-loops blocks one DNA strand from N_3_-kethoxal (green dots) labeling, resulting in an unbalanced spKAS-seq read density on two DNA strands. Pull-down was performed under a denaturing condition to ensure capturing of only the N_3_-kethoxal–modified strand. (**B**) Snapshots of two representative genomic loci showing the difference between traditional KAS-seq and spKAS-seq profiles. (**C** and **D**) Volcano plots showing an unbalanced read density between two DNA strands detected by traditional KAS-seq (C) and spKAS-seq (D). Equally sized 2-kb bins of the hg19 reference genome were used for analysis. chr1, chromosome 1.

Existing R-loop mapping approaches, either by S9.6 or RNase H, all appear to target the RNA-DNA hybrid duplexes. Meanwhile, another unique structural property of R-loops, namely, the exposure of ssDNA on only one DNA strand, has not been used for R-loop mapping. Taking advantage of N_3_-kethoxal to react with guanines that do not form Watson-Crick base pairing, we have developed KAS-seq to profile ssDNA in situ using as few as 1000 cells (*32*). N_3_-kethoxal reacts with both DNA strands in transcription bubbles when nascent RNA does not form base pairing interactions with its DNA template, while in R-loops, it only labels the exposed DNA strand (Fig. 1A). We envision that if we can specifically enrich the N_3_-kethoxal–modified DNA strand and apply strand-specific library construction, then we can readily identify R-loops with low-input materials by detecting the imbalanced ssDNA read numbers mapped to two DNA strands (Fig. 1A).

In spKAS-seq, we applied two modifications to the original KAS-seq protocol to ensure strand specificity. First, we performed the enrichment step under denaturing conditions by incubating the streptavidin beads in a 100 mM sodium hydroxide (NaOH) solution (Fig. 1A). This step washes away the unlabeled DNA strand, preventing them from being captured by hybridizing to the N_3_-kethoxal–modified strand. Second, we adopted an ssDNA ligation–based library construction to ensure strand-specific DNA amplification.

To demonstrate the robustness of the protocol, we performed spKAS-seq in three different human cell lines [human embryonic kidney 293T (HEK293T), HepG2, and K562]. spKAS-seq data exhibit a strong correlation between replicates in all three cell lines (fig. S1A) and achieve an enrichment efficiency similar to the original KAS-seq (fig. S1, B to D). KAS-seq and spKAS-seq show similar patterns at the gene-coding regions (fig. S1E); however, the numbers of spKAS-seq reads on template and nontemplate DNA strands at a given locus can be evidently different (Fig. 1B). Statistically, we calculated the number of 2-kb genomic bins that have different read density between two DNA strands in HEK293T cells. More than 36,000 bins show notable spKAS-seq read density difference, while only around 2900 bins show such difference in KAS-seq (Fig. 1, C and D). In KAS-seq data, most of these bins have a read density of no more than 20, which is much lower compared to the read density of such bins in spKAS-seq data (fig. S1F). A higher number of spKAS-seq reads were mapped to the nontemplate DNA strand, which is consistent with the orientation of transcription (fig. S2). These findings collectively suggest that spKAS-seq can specifically enrich the N_3_-kethoxal–modified ssDNA and reveal asymmetric ssDNA distribution on two DNA strands. Because spKAS-seq was performed using a heterogeneous cell population, spKAS-seq data at a given locus may include a mixture of signals deriving from transcription bubbles and R-loops.

### spKAS-seq identifies native R-loops with low-input materials

**Fig. 2.**
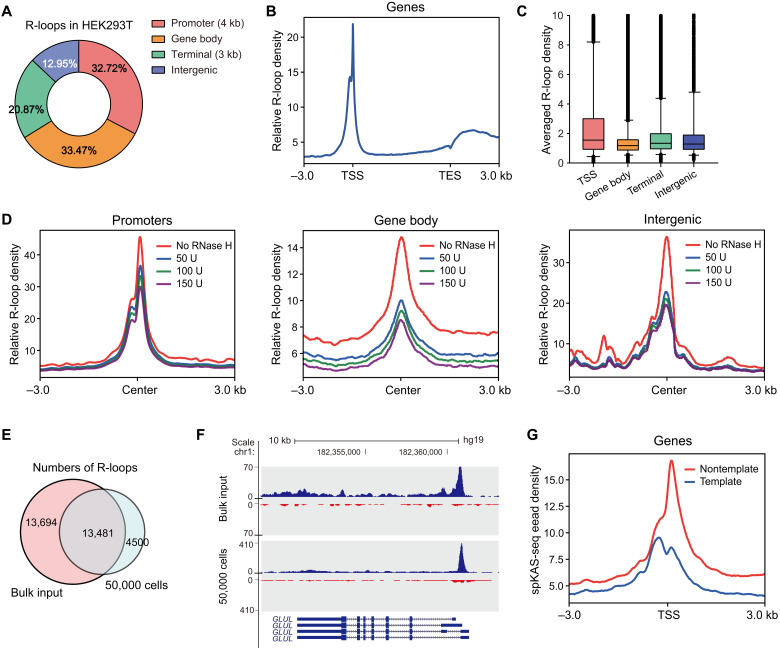
spKAS-seq maps cellular R-loops with low-input materials. (**A**) The genomic distribution of R-loops identified by spKAS-seq in HEK293T cells. (**B**) The metagene profile of R-loops at gene-coding regions in HEK293T cells was revealed by spKAS-seq. Relative R-loop density was calculated as the spKAS-seq read density difference between the template and nontemplate strands. (**C**) The R-loop density at different genomic locations in HEK293T cells. (**D**) Metagene analysis of promoter, gene body, and intergenic R-loop levels in HEK293T cells was treated by different dosages of RNase H. (**E**) The overlap between R-loops detected by spKAS-seq using 50,000 and bulk HEK293T cells. (**F**) A snapshot showing spKAS-seq signals generated by 50,000 and bulk HEK293T cells on a representative R-loop region. (**G**) spKAS-seq reads density on template and nontemplate strands of R-loop regions were detected using 50,000 HEK293T cells.

We next defined R-loops as regions with notable spKAS-seq read density difference between template and nontemplate strands (see Materials and Methods) and defined the absolute value of the read density difference as R-loop density. R-loops detected by spKAS-seq exhibit two- to threefold read density difference between two DNA strands on average (fig. S3A) and show a strong correlation between replicates (fig. S3B). Consistent with results from other R-loop mapping approaches, R-loops detected by spKAS-seq are enriched around TSSs in all three tested cell lines (Fig. 2, A and B, and fig. S3C). R-loops are more enriched at promoter regions compared to KAS-seq peaks (fig. S3D). R-loop signals were also observed on gene bodies and transcription end sites (TESs) (Fig. 2, A and B, and fig. S3C). R-loops around TSS show higher strength than those at other genomic locations (Fig. 2C), and most R-loops are not longer than 2 kb in length (fig. S3E).

We then examined whether R-loops identified by spKAS-seq are sensitive to RNase H digestion. We permeabilized HEK293T cells and treated the cells with RNase H at different dosages (50, 100, or 150 U). RNase H digests the RNA strand that pairs with the template strand DNA within the RNA-DNA hybrid. This should lead to the exposure of the template strand for N_3_-kethoxal labeling and/or the annealing of two DNA strands, resulting in ssDNA signal increase on the template strand and/or ssDNA signal decrease on the nontemplate strand. RNase H treatment abolished the spKAS-seq read density difference between two DNA strands on both TSS and gene body (fig. S4A). Statistically, R-loop density from spKAS-seq showed a dose-dependent reduction upon RNase H treatment at promoter, gene body, and intergenic regions (Fig. 2D), giving rise to a dose-dependent R-loop number decrease across the genome (fig. S4, B and C). Notably, RNase H did not eliminate all R-loop signals, potentially because of the presence of a subset of R-loops that are resistant to RNase H treatment (*25*) or limited digestion efficiency in permeabilized cells.

While most R-loop mapping approaches require bulk input materials because of the nature of antibody-based pull-down, spKAS-seq maintains a high sensitivity for R-loop detection when using low-input materials. Using 50,000 HEK293T cells, spKAS-seq detected 17,981 R-loops, with 75% of them overlapping with R-loops detected using bulk cells (Fig. 2, E and F). These R-loops show clear read density difference between two strands (Fig. 2G), enrich assay for transposase-accessible chromatin using sequencing (ATAC-seq) signals, and display high occupancy of Pol II and histone markers for active transcription (fig. S5).

Apart from R-loops at gene-coding loci, spKAS-seq also detects R-loops at regulatory elements and other intergenic regions. In HEK293T cells, for example, spKAS-seq detects 1671 R-loops at enhancers, many of which overlap with R-loops identified by R-ChIP and DRIP-seq (fig. S6, A and B). These enhancers show stronger ATAC-seq and H3K27ac binding signals than other active enhancers (fig. S6, C and D). Genes close to R-loop–positive enhancers show a higher level of merged spKAS-seq signals (fig. S6E) and RNA level (fig. S6F). R-loops were also detected in 180 of 606 annotated tRNA gene loci (fig. S6, G and H). The unevenness of spKAS-seq read density was also observed at some telomere regions (fig. S6I), which may be attributed to R-loops generated by telomere repeat-containing RNA or telomere DNA displacement (*34*, *35*). Note that noncanonical DNA structures other than R-loops, such as triple-strand DNA (H-DNA), may also expose ssDNA on only one strand and contribute to a small portion of spKAS-seq signals.

### Comparison between spKAS-seq and other R-loop mapping technologies

**Fig. 3.**
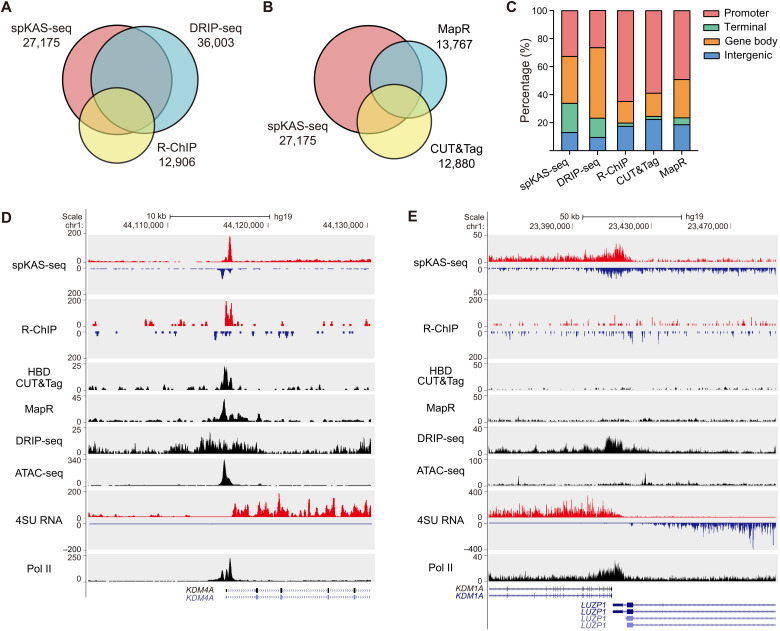
Benchmarking spKAS-seq with other R-loop mapping methods. (**A**) The overlap between R-loops detected by spKAS-seq, DRIP-seq, and R–ChIP in HEK293T cells. (**B**) The overlap between R-loops detected by spKAS-seq, His-tagged HBD CUT&Tag, and MapR in HEK293T cells. (**C**) The percentage of R-loops on denoted genomic locations revealed by spKAS-seq, DRIP-seq, R-ChIP, His-tagged HBD CUT&Tag, and MapR. (**D**) A snapshot from the UCSC Genome Browser showing an R-loop at the 5′ end of the KDM4A gene. This is an open chromatin region (as revealed by ATAC-seq), and all five methods could detect the corresponding R-loop. (**E**) A snapshot from the UCSC Genome Browser showing an R-loop in the transcription termination region of the actively transcribing LUZP1 gene, where ATAC-seq signal was not enriched. Only spKAS-seq and DRIP-seq show signals in this region. 4SU, 4-thiouridine.

We next comprehensively compared spKAS-seq with other R-loop mapping methods, including DRIP-seq (*11*), R-ChIP (*29*), MapR (*30*), and cleavage under targets and tagmentation (CUT&Tag), based on an engineered hybrid binding domain (HBD) of RNase H1 (*31*). These methods involve different enrichment strategies (S9.6 and RNase H) performed ex vivo (DRIP-seq) or in situ (R-ChIP, MapR, and HBD CUT&Tag). R-loops detected by different assays in HEK293T cells show decent overlap, with spKAS-seq and DRIP-seq detecting much more R-loops than the other methods (Fig. 3, A and B). spKAS-seq captures RNA-DNA hybrids in gene bodies and at transcription end site (TES), while R-ChIP, MapR, and CUT&Tag enrich R-loops mostly at promoter regions (Fig. 3C). DRIP-seq exhibits a lower resolution than the other assays (Fig. 3, D and E, and fig. S7, A to C), leading to a substantially higher R-loop length and genomic coverage (fig. S7, D and E).

Because R-ChIP, MapR, and HBD CUT&Tag are all based on the binding to RNA-DNA duplex by RNase H, the disparities between results from these assays and from spKAS-seq could partially attribute to the preference of RNase H to certain R-loops (*25*). In addition, because MapR and HBD CUT&Tag rely on chromatin digestion by micrococcal nuclease (MNase) or Tn5 transposase (*30*, *31*), their R-loop capture efficiency may interfere with chromatin accessibility. In HEK293T cells, we calculated the fraction of R-loops detected by different methods that overlap with ATAC-seq peaks. In contrast to spKAS-seq and DRIP-seq that show around 40% overlap, more than 60% of MapR, HDB CUT&Tag, and R-ChIP peaks overlap with ATAC-seq peaks (fig. S7F). While all methods can detect R-loop at open chromatin regions (Fig. 3D), only spKAS-seq and DRIP-seq detect R-loops at transcription termination regions of actively transcribing genes, where ATAC-seq peak was not present (Fig. 3E and fig. S7G). These findings provide a mechanistic explanation for different R-loop profiles observed between in vivo and ex vivo capture protocols using the same antibody (*31*). Therefore, spKAS-seq maps R-loops in vivo while maintaining the high-sensitivity and unbiased features shared by ex vivo approaches.

### spKAS-seq detects R-loop dynamics in response to transcription perturbations

**Fig. 4.**
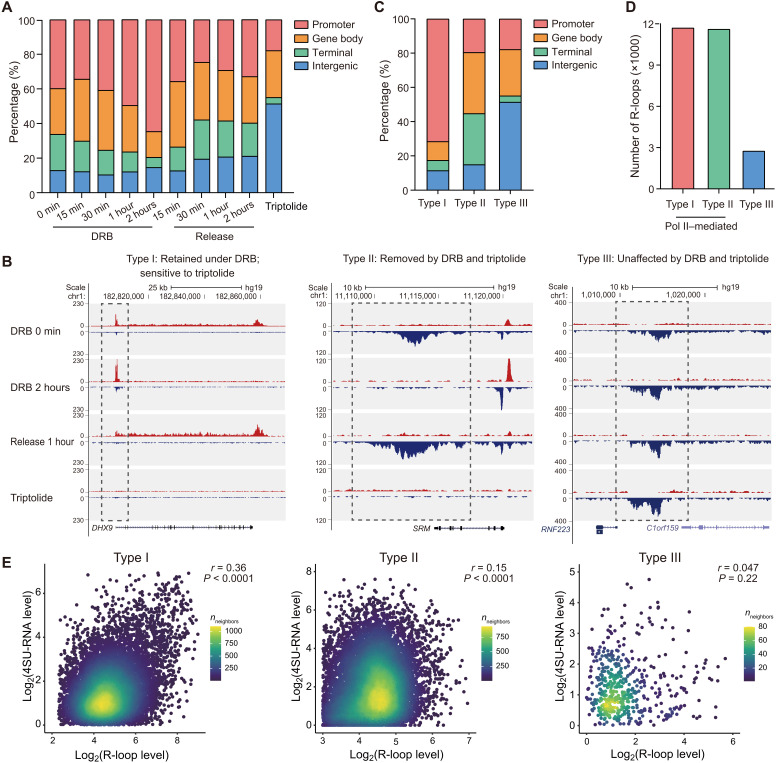
R-loop levels could respond to transcription perturbations and correlate with transcription activity. (**A**) The percentage of R-loops at different genomic locations at denoted time points after DRB treatment, DRB removal, or triptolide treatment in HEK293T cells. (**B**) Three types of R-loops were identified by spKAS-seq. Representative snapshots of spKAS-seq at denoted time points after DRB treatment, DRB removal, or triptolide treatment were shown. (**C**) The distribution of three types of R-loops at different genomic locations. (**D**) The amounts of three types of R-loops were identified by spKAS-seq. (**E**) The correlation between R-loops and nascent RNA (4SU RNA) levels at the corresponding genomic loci. Correlations for each type of R-loop were plotted separately, with the *r* values denoting Pearson correlation coefficients. *P* values were calculated using *t* distribution.

Because R-loop formation is coupled with transcription, R-loops at coding regions can dynamically respond to transcription perturbations. Meanwhile, R-loops at different genomic locations may have varied impacts on transcription regulation (*9*, *36*, *37*). To better understand the interplay between R-loop and transcription, we performed spKAS-seq at various time points after treating HEK293T cells with DRB (5,6-dichloro-1-β-ribofuranosylbenzimidazole), which is known to reversibly inhibit Pol II elongation and induce Pol II pausing at TSS (*38*). R-loop density at TSS gradually increased from 15 min to 2 hours after DRB treatment, while R-loop density at the gene body and termination regions gradually reduced (Fig. 4A and fig. S8A). After the 2-hour treatment, the release of inhibition by removing DRB reversed this trend (Fig. 4A and fig. S8, B to D). As a control experiment, treating cells with triptolide that impedes Pol II recruitment to TSS abolished most R-loop signals at the gene-coding regions (fig. S8, A, C, and D). Thus, spKAS-seq detects R-loop dynamics within 15-min intervals.

We then classified R-loops into three types according to their susceptibility to DRB and triptolide: type I, retained under DRB but abolished by triptolide; type II, sensitive to DRB and triptolide; and type III, impervious to DRB and triptolide. As expected, type I R-loops are enriched at promoters and exhibit the strongest signals, whereas type II R-loops show a lower intensity and primarily localize at the gene bodies and transcription termination regions (Fig. 4, B and C, and fig. S8E). Type II R-loops may also include RNA-DNA hybrids formed within transcription bubbles during active transcription. Type III R-loops are less abundant and are mostly intergenic (Fig. 4, B to D). All three types of R-loops show a dose-dependent response to RNase H treatment (fig. S8F). Type III R-loops include R-loops that are associated with nascent RNA transcribed by RNA Pol I and Pol III (such as those at tRNA loci) and may include other potential noncanonical DNA structures.

We next studied how different types of R-loops interplay with transcription by plotting the correlation between R-loop strength and nascent RNA level at the same locus. Both type I and type II R-loops positively correlate to nascent transcription, with type I R-loops showing a stronger association (Fig. 4E). Many type III R-loops are not located at transcription units and are, thus, not correlated with nascent RNA levels (Fig. 4E).

### R-loops are associated with chromatin-binding RBPs

Although many R-loops are formed cotranscriptionally, certain R-loops have been proposed to exert long-term effects by attracting or repelling chromatin remodeler proteins (*12*–*14*). However, this effect was only observed on a limited number of TFs at specific R-loop loci. Many RBPs are ubiquitously associated with chromatin to affect transcription and RNA processing (*39*, *40*), but factors that lead to the preferences of RBPs for certain DNA targets remain elusive.

**Fig. 5.**
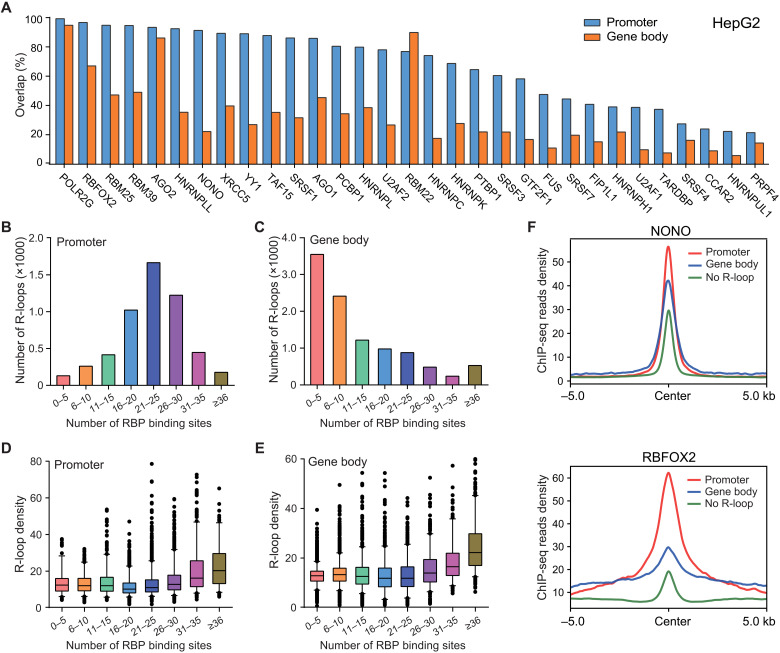
R-loops are associated with RBPs on the chromatin. (**A**) The percentages of R-loops that overlap with the ChIP-seq peaks of each denoted RBP at promoters and gene bodies in HepG2 cells. (**B** and **C**) The numbers of promoter (B) and gene body (C) R-loops that overlap with different numbers of RBP ChIP-seq peaks in HepG2 cells. (**D** and **E**) The relationship between the number of RBP ChIP-seq peaks and the strength of promoter (D) and gene body R-loops (E) in HepG2 cells. (**F**) The strength of Non-POU domain-containing octamer-binding protein (NONO) (top) and RNA binding fox-1 homolog 2 (RBFOX2) (bottom) ChIP-seq peaks that overlap with promoter R-loops, gene body R-loops, and R-loop–negative regions in HepG2 cells. For boxplots in (D) and (E), the 5th to 95th percentile of data points were plotted, with the center line depicting the median and the box limits showing the upper and lower quartiles.

Because RBPs have an intrinsic ability to bind single-stranded nucleic acids, we envision that R-loop may contribute to the binding of RBPs to certain chromatin regions. We studied the association between RBPs and R-loops by plotting the percentage of R-loops that overlap with RBP ChIP-seq peaks in HepG2 and K562 cells. Promoter R-loops show a strong overall association with RBPs. The ChIP-seq peaks of 21 RBPs in HepG2 cells and 14 RBPs in K562 cells overlap with more than 50% promoter R-loops (Fig. 5A and fig. S9A). In contrast, only DNA-directed RNA polymerase II subunit G (POLR2G), RNA binding fox-1 homolog 2 (RBFOX2), RNA-binding motif protein 22 (RBM22), and argonaute-2 (AGO2) show high overlap with R-loops in the gene body (Fig. 5A and fig. S9A). Consistently, promoter R-loops are bound by more RBPs on average (Fig. 5, B to C, and fig. S9, B and C), corroborating a previous observation that promoters are RBP binding hotspots (*39*). The number of RBP ChIP-seq peaks on R-loops correlates with R-loop strength at both promoter and gene body (Fig. 5, D and E, and fig. S9, D and E), potentially because RBP binding causes more active transcription (*39*) or suggesting that certain RBPs could stabilize R-loops. We then compared the strength of RBP ChIP-seq peaks on R-loop–positive and R-loop–negative regions in HepG2 and K562 cells. Many RBPs have a higher binding density on R-loop–positive ChIP-seq peaks than on the other ChIP-seq peaks (Fig. 5, F and G, and fig. S10, A and B), suggesting an association between R-loops and RBP binding on the chromatin.

## DISCUSSION

We introduce spKAS-seq for R-loop mapping by combining biotin pull-down under a denaturing condition with strand-specific ssDNA library construction. spKAS-seq identifies R-loops by measuring the read density difference between the template and nontemplate strands (see Materials and Methods) rather than direct peak calling. This is distinct from other approaches that involve antibodies or recombinant proteins to recognize RNA-DNA duplexes, which may inadvertently perturb R-loop and prefer certain chromatin regions. spKAS-seq is capable of providing information on the displaced strand, which is not available in MapR and CUT&Tag. Taking full advantage of efficient N_3_-kethoxal labeling, spKAS-seq works with 50,000 live cells, broadening its application in biological systems that involve rare cell populations (such as primary cells) and clinical samples.

Results from spKAS-seq suggest that some previously identified transcription bubbles using KAS-seq include a portion of RNA-DNA duplex. This should not compromise both spKAS-seq and the original KAS-seq in measuring the transcription activity, as it detects all ssDNAs that include all critical information about transcription initiation, elongation, and termination. The original KAS-seq could not identify R-loops because of the lack of strand information. spKAS-seq may also be used to map transient R-loop structures generated by the CRISPR-Cas machinery (*41*) and other noncanonical DNA structures.

Different from R-ChIP, MapR, and CUT&Tag, spKAS-seq detects an appreciable level of RNA-DNA hybrids within gene bodies. This observation seems to corroborate DRIP-seq; however, recent findings suggest that most DRIP-seq signals within gene bodies appear to derive from dsRNA rather than R-loops (*42*, *43*). Note that the presence of spKAS-seq signals and their sensitivity to RNase H treatment indicate the presence of R-loops within gene bodies. However, these RNA-DNA hybrids within gene bodies may include not only regular R-loops but also RNA-DNA hybrids between the template DNA strand and nascent RNA within transcription bubbles. Future investigations are required to further differentiate these different RNA-DNA hybrids at gene bodies. Apart from R-loops, other noncanonical DNA structures in the genome may also expose only one DNA strand, which could complicate accurate R-loop quantification and pose a potential limitation of spKAS-seq.

By correlating R-loops with a large RBP ChIP-seq dataset, we showed that R-loops can be hotspots for RBP binding on the chromatin. High RBP binding was observed on R-loops, suggesting that RBPs could bind either ssDNA or the RNA-DNA duplex. Many RBPs, including KH domain family proteins and zinc fingers, were shown to have ssDNA binding activity (*44*). Protein pull-down by RNA-DNA hybrids followed by mass spectrometry confirmed that many RBPs can bind to duplex structures (*45*). The exact component(s) to which each RBP bind is still an open question that requires biochemical characterizations in the future.

## MATERIALS AND METHODS

### Cell culture

HEK293T and HepG2 cells were purchased from the American Type Culture Collection (ATCC) (CRL11268 for HEK293T and HB8065 for HepG2) and were cultured in Dulbecco’s modified Eagle’s medium (Gibco, 11995) supplemented with 10% (v/v) fetal bovine serum (Gibco) and 1% penicillin and streptomycin (Gibco, 10378) and grown at 37°C with 5% CO_2_. K562 cells were purchased from ATCC (CCL243) and were cultured in RPMI 1640 (Gibco, 11875) supplemented with 10% (v/v) fetal bovine serum (Gibco) and 1% penicillin and streptomycin (Gibco) and grown at 37°C with 5% CO_2_. All cell lines were routinely checked to be free of mycoplasma.

### spKAS-seq

Treat cells with 5 mM N_3_-kethoxal dissolved in their culture medium for 10 min at 37°C with 5% CO_2_. After labeling, harvest cells for genomic DNA isolation using the PureLink Genomic DNA Mini Kit (Thermo Fisher Scientific, K182002). Mix around 2 μg of purified genomic DNA with 5 μl of 20 mM dibenzocyclooctyne-PEG_4_-biotin conjugate (Sigma-Aldrich, 760749) and 10 μl of 10× phosphate-buffered saline (PBS) and adjust the total volume to 100 μl with 25 mM K_3_BO_3_. Gently shake the mixture at 37°C for 1.5 hours, then add 5 μl of RNase A (Thermo Fisher Scientific, 12091039), and shake the mixture for another 5 min at 37°C. After the reaction, purify DNA using the DNA Clean & Concentrator-5 kit (Zymo Research, D4013) and fragment DNA to 150 to 350 base pairs (bp) by sonicating 30 cycles at 30-s on/30-s off setting using the Diagenode Bioruptor Pico.

Save 5% of the sonicated DNA as input and use the rest for enrichment with 10 μl of Dynabeads MyOne Streptavidin C1 (Thermo Fisher Scientific, 65001). Wash beads with 1× B&W buffer [5 mM tris-HCl (pH 7.4), 0.5 mM EDTA, 1 M NaCl, and 0.05% Tween 20], then resuspend beads in 95 μl of 2× B&W buffer, and mix beads with sonicated DNA. Perform binding at room temperature for 15 min. Wash beads once with 1× B&W buffer, twice with 100 mM NaOH solution to denature the dsDNA and remove the DNA strands that are not labeled by N_3_-kethoxal, and once again with 1× B&W buffer. Elute DNA from washed beads in 10 μl of H_2_O by heating the beads at 95°C for 10 min. Take enriched DNA and the corresponding input for library construction using the Accel-NGS Methyl-Seq DNA Library Kit (Swift, 30024). Sequence libraries on Illumina platforms with at least 60 million reads per library. spKAS-seq using 50,000 cells was performed by following the same procedure with the following changes: (i) Isolate DNA using a Quick genomic DNA mini plus kit (Zymo Research, D4068), (ii) use all genomic DNA for biotinylation, (iii) scale down the biotinylation reaction to a volume of 50 μl, and (iv) use 5 μl of Dynabeads MyOne Streptavidin C1 for enrichment.

### RNase H treatment for spKAS-seq

One million freshly collected HEK293T cells were resuspended in 1 ml of ice-cold lysis buffer [20 mM Hepes (pH 7.9), 10 mM KCl, 1 mM MgCl_2_, 0.1% Triton X-100, and 20% glycerol] and incubated on ice for 10 min. The nuclei-containing pellets were then collected by centrifugation at 2500*g* and washed once with 500 μl of ice-cold wash buffer [20 mM Hepes (pH 7.9), 75 mM KCl, 3 mM MgCl_2_, 0.5 mM spermidine, and 0.1% bovine serum albumin]. Washed nuclei were resuspended into 500 μl of wash buffer and split into halves, with one-half supplemented with 30 μl of RNase H (New England Biolabs, M0297L) and another half with 30 μl of water as a control. The mixtures were then incubated at 37°C for 1 hour with a gentle shake. The nuclei were then collected by centrifugation and then resuspended into 200 μl of wash buffer containing 2 mM N_3_-kethoxal. The labeling was allowed for 10 min at 37°C before nuclei were collected by centrifugation and used for total DNA isolation using the PureLink Genomic DNA Mini Kit (Thermo Fisher Scientific, K182002).

### DRB and triptolide treatment

For DRB treatment, HEK293T cells were incubated in media that contains 100 μM DRB (Sigma-Aldrich, D1916) for 0, 15, 30, 60, and 120 min before N_3_-kethoxal labeling. For the DRB release experiment, cells were treated for 120 min with DRB first. Then, we removed the DRB-containing media, washed cells once with Dulbecco’s PBS, and incubated cells in fresh media for 15, 30, 60, and 120 min before N_3_-kethoxal labeling. For triptolide treatment, cells were incubated for 2 hours in media that contains 1 μM triptolide (Sigma-Aldrich, T3652) before being used for spKAS-seq.

### spKAS-seq data processing

All spKAS-seq data in this study were performed with two replicates. No sample was excluded for analysis. Trim Galore (*46*) was used to remove low-qualified bases and adapter-containing reads from raw spKAS-seq data. Trimmed reads shorter than a length of 30 bp were discarded, and the rest were aligned to the reference genome (hg19) using Bowtie2 (v2.3.3.1) (*47*) under default parameters. Sam files were subsequently converted and sorted to binary alignment map (BAM) files using samtools sort (v1.9) (*48*). Duplicated reads were removed using Picard MarkDuplicates (v1.141). For paired-end spKAS-seq data, SAMtoBED.py was used to combine “properly paired” alignments into a single-bed interval. For single-end spKAS-seq data, deduplicated unique mapped reads were extended to 150 bp to match the average length of DNA fragments using the awk command. Browser extended data (BED) files were converted to BedGraph files using bedtools genomecov (*49*). BedGraph files were then converted to BigWig files using bedGraphToBigWig from University of California, Santa Cruz (UCSC) precompiled utilities. Shell scripts for spKAS-seq data mapping and quality control are provided in GitHub (https://github.com/Ruitulyu/KAS-pipe2) (*50*). All the metagene profile plots and heatmaps were generated using deepTools plotProfile and plotHeatmap (*51*).

### spKAS-seq peaks calling

We used MACS2 (*52*) to call spKAS-seq peaks (macs2 callpeak -t spKAS-seq_IP.bed -c spKAS-seq_Input.bed -n spKAS-seq_peaks.bed --broad -g hs --broad-cutoff 0.01 -q 0.01). As spKAS-seq shows broad peaks on gene bodies, MACS2 was run to call broad peaks by linking nearby highly enriched regions (--broad) under default parameters.

### RNA sequencing data processing

Clean raw RNA sequencing (RNA-seq) reads were mapped to the reference genome (hg19) with HISAT2 (*53*) under default settings. The expression level of each gene was quantified as FPKM values with the Fragments per kilo base of transcript per million mapped reads (FPKM)_count.pl script in the RSeQC package (*54*). Genes with FPKM values higher than 0.5 were defined as expressed genes. Expressed genes were ranked on the basis of their FPKM values, with the top 2000 defined as highly expressed genes, 2000 genes in the middle defined as medium expressed genes, and the bottom 2000 defined as lowly expressed genes, and genes with FPKM values lower than 0.5 were defined as silent genes. R-loop density calculated by spKAS-seq, DRIP-seq, and R-ChIP were then plotted on these four groups, respectively.

### ATAC-seq and ChIP-seq data processing

The preprocessing and mapping procedures for ATAC-seq and ChIP-seq data are the same as indicated in the “spKAS-seq data processing” section. MACS14 was used to call ChIP-seq and ATAC-seq peaks using default parameters (macs14 -t RBP.bed -c Input -n RBP_peaks -p 1e-7). Enriched consensus motifs on RBP binding sites were analyzed by HOMER (*55*).

### Definition of R-loops by spKAS-seq

Two spKAS-seq replicates were used for R-loops identification. Deduplicated mapped spKAS-seq reads were split into “plus” and “minus” strands. Reads on these two strands were converted to BedGraph and BigWig files as described above in the “spKAS-seq data processing” section. The hg19 genome was then divided into 500-bp bins with a 250-bp overlap using bedtools makewindows. Bins overlapped with the human genome blacklist [the encyclopedia of DNA elements (ENCODE)] were excluded from downstream analysis. The remaining bins with at least 250-bp overlap with KAS-seq peaks were filtered as the candidate bins for R-loop definition. multiBigwigSummary from deepTools was then applied to calculate the averaged read density on plus and minus strands of the candidate bins. In KAS-seq, most bins that show asymmetric read numbers between two strands have a read density of 20 or less. Therefore, in spKAS-seq, bins with averaged read density higher than 20 and significantly uneven reads distribution on two strands [*q* ≤ 0.05, log_2_(plus/minus) ≥ 1 or log_2_(plus/minus) ≤ −1) were identified as R-loops bins. Overlapped R-loop bins were then merged using bedtools merge and defined as R-loops. R-loop density was calculated as the spKAS-seq read density difference (absolute values) between plus and minus strands.

### Correlation analysis

deepTools multiBigwigSummary was used to calculate the averaged read density within equally sized 5-kb bins of the entire genome. Bins that overlapped with the human genome ENCODE blacklist were excluded, and only bins that overlapped with spKAS-seq peaks or R-loops were kept for correlation analysis. For correlation analysis between R-loops, the expression levels were defined using RNA-seq data. The calculations of Pearson correlation coefficients (*r* values) and the corresponding *P* values were performed using R scripts.
