# Supplementary material for: spKAS-seq reveals R-loop dynamics using low-input materials by detecting single-stranded DNA with strand specificity
Source: Sci Adv. 2022 Nov 30;8(48):eabq2166. doi: 10.1126/sciadv.abq2166 (PMC9710868; doi:10.1126/sciadv.abq2166)
Supplement: Supplementary file 1 — Figs. S1 to S10 [file sciadv.abq2166_sm.pdf]

Science Advances
AAAS

Supplementary Materials for

spKAS-seq reveals R-loop dynamics using low-input materials by detecting single-stranded DNA with strand specificity

Tong Wu *et al*.

Corresponding author: Chuan He, chuanhe@uchicago.edu

Sci. Adv. **8**, eabq2166 (2022)
DOI: 10.1126/sciadv.abq2166

The PDF file includes:

Figs. S1 to S10
Legends for data S1 and S2

Other Supplementary Material for this manuscript includes the following:

Data S1 and S2

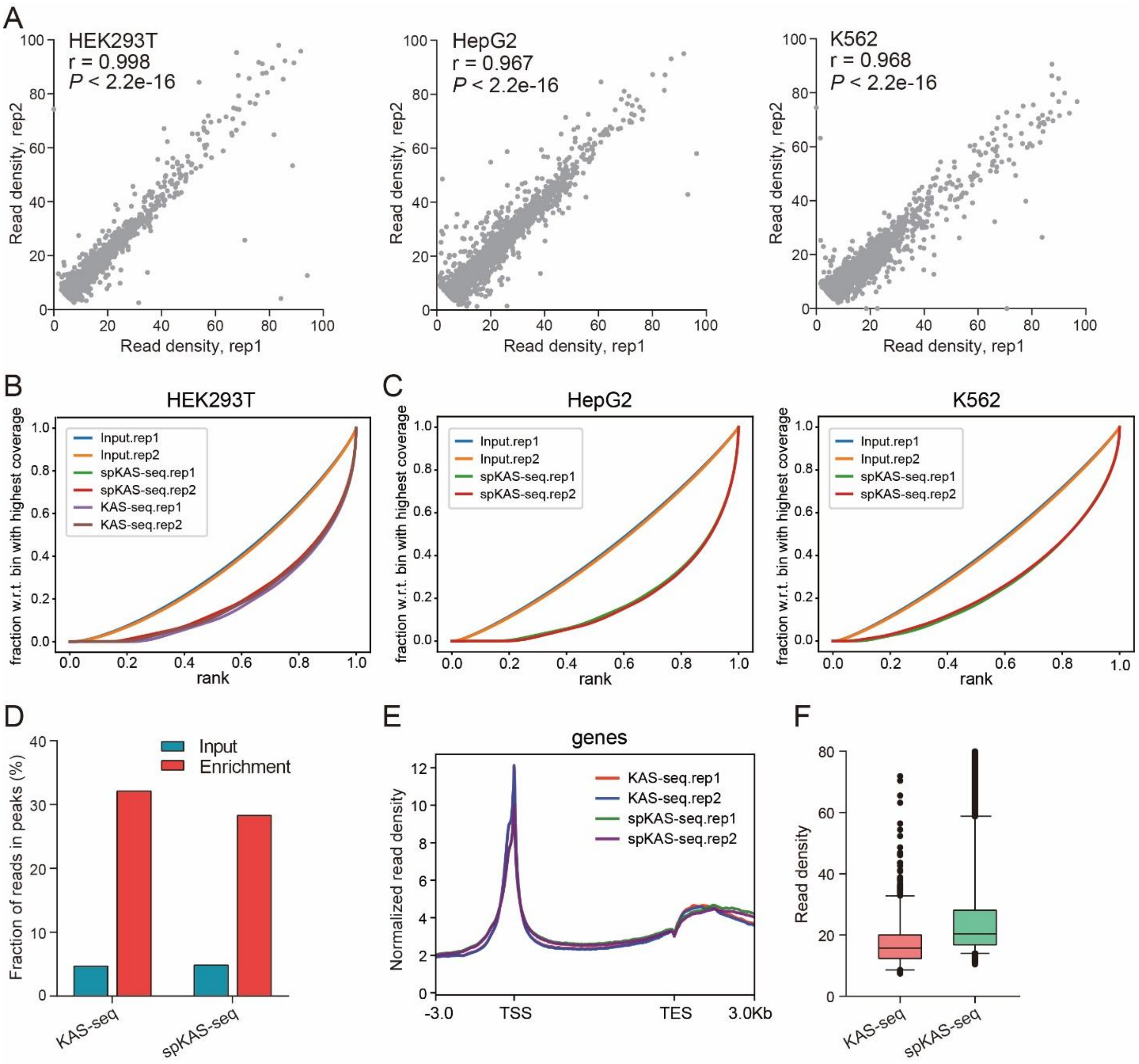

Figure S1. Quality control of spKAS-seq. (**A**) Pearson correlation between two spKAS-seq technical replicates in HEK293T, HepG2, and K562 cells, respectively. The x- and y-axis show spKAS-seq read density in randomly-sized 5 kb genomic bins. *P* values were determined using t-distribution. (**B**) The fingerprint plot of spKAS-seq in comparison with regular KAS-seq in HEK293T cells. (**C**) Fingerprint plots of spKAS-seq in HepG2 and K562 cells. (**D**) The fraction of reads in peaks for regular KAS-seq, spKAS-seq and their corresponding input libraries in HEK293T cells. (**E**) Normalized KAS-seq and spKAS-seq reads density at gene coding regions in HEK293T cells. (**F**) The read density in the genomic bins showing asymmetric KAS-seq and spKAS-seq signals between two DNA strands in HEK293T cells.

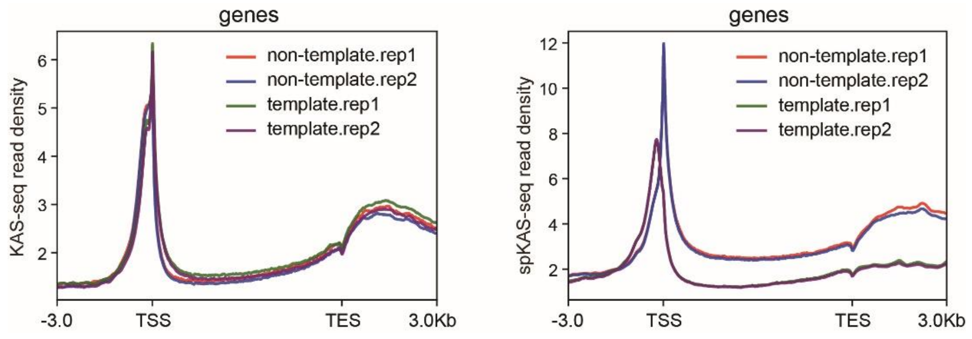

Figure S2. The reads distribution of KAS-seq (left) and spKAS-seq (right) on template and non-template strands. spKAS-seq (right) reveals distinct read density between two DNA strands. The original KAS-seq (left) does not identify this read density difference.

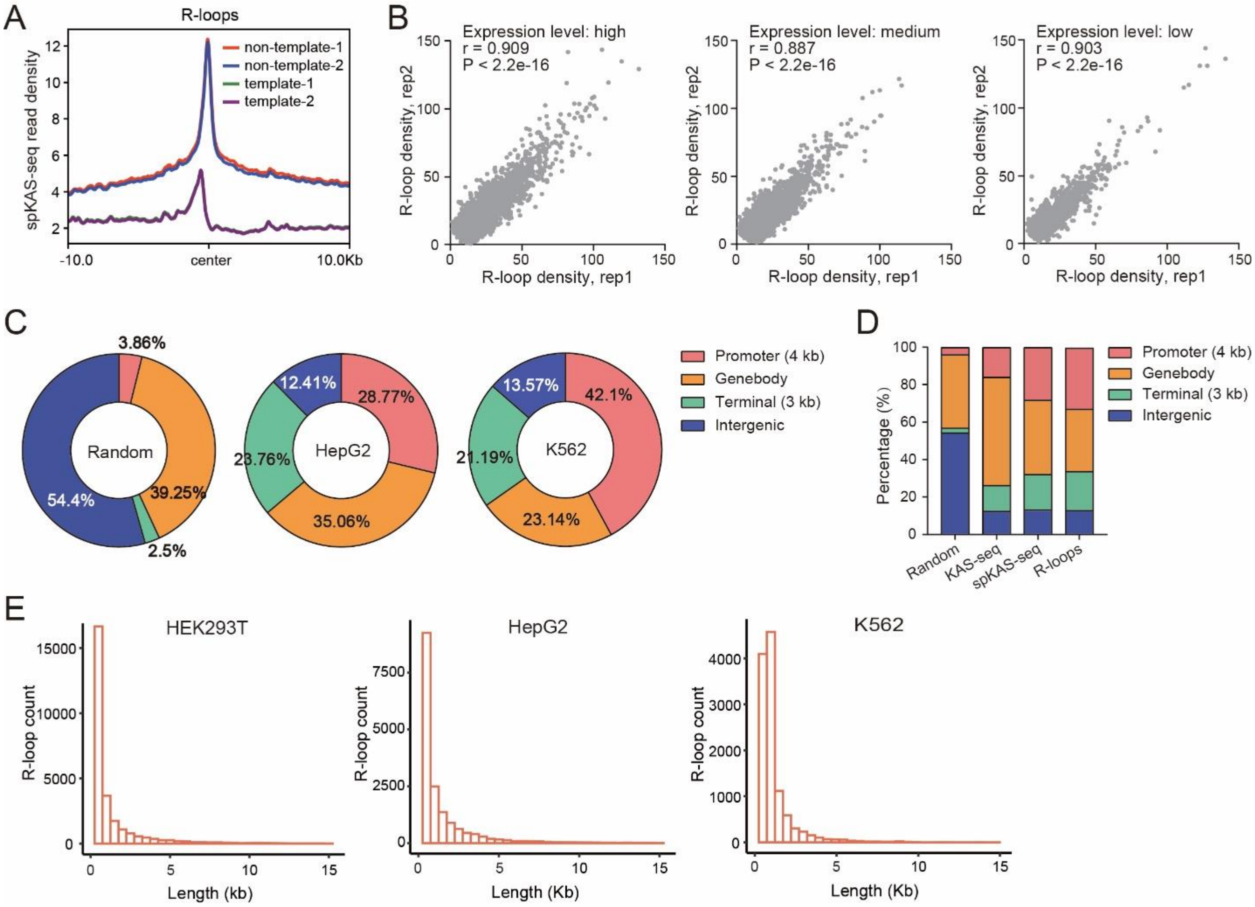

Figure S3. Features of R-loops identified by spKAS-seq. (**A**) spKAS-seq read density on template and non-template strands of promoter R-loop regions in HEK293T cells. (**B**) Correlation between R-loops identified by two spKAS-seq technical replicates at genes with different expression levels in HEK293T cells. Genes were divided into three groups according to their mRNA abundance revealed by RNA-seq data. *P* values were determined using t-distribution. (**C**) The genomic distribution of R-loops in HepG2 and K562 cells. The distribution of randomly generated regions with the same length and number of R-loops was shown for comparison. (**D**) The genomic distribution of KAS-seq peaks, spKAS-seq peaks, and R-loops identified by spKAS-seq in HEK293T cells. (**E**) The distribution of the R-loop length in HEK293T, HepG2, and K562 cells.

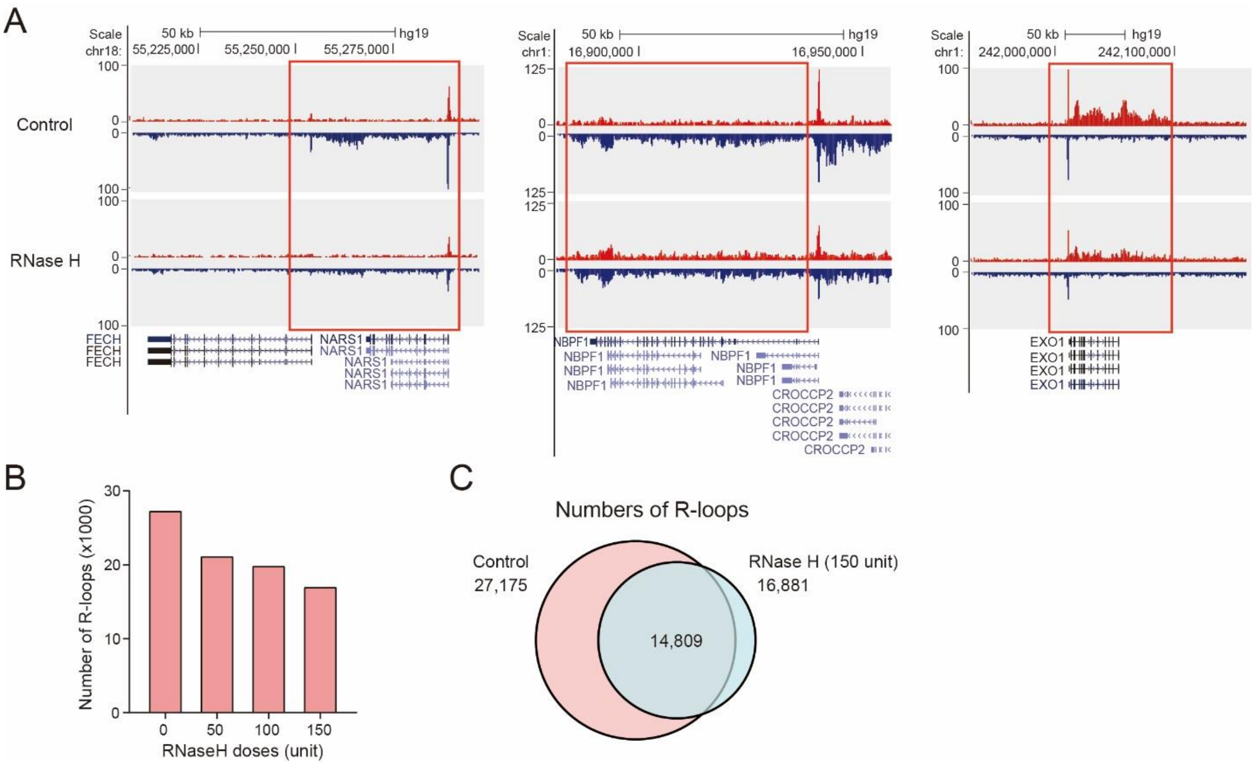

Figure S4. R-loops detected by spKAS-seq are sensitive to RNase H treatment. (**A**) Snapshots of spKAS-seq signals before and after RNase H (150 units) treatment at three representative R-loop loci in HEK293T cells. Regions that respond to RNaseH treatment were circled in red. (**B**) The numbers of R-loops detected by spKAS-seq in HEK293T cells treated by different dosages of RNase H. (**C**) The overlap between R-loops detected by spKAS-seq in control and RNase H-treated HEK293T cells.

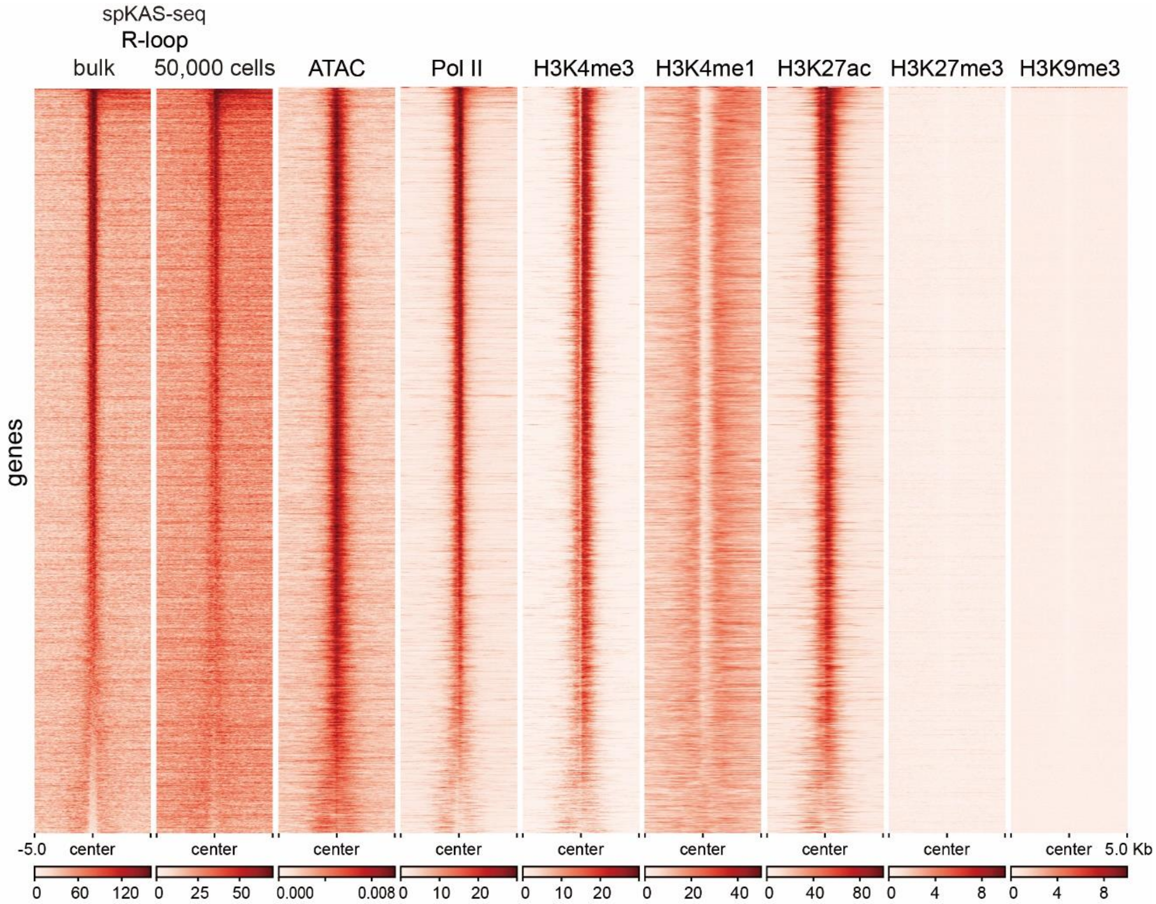

Figure S5. ATAC-seq, Pol II, and histone modifications signals on R-loops detected by spKAS-seq in HEK293T cells. R-loops detected by spKAS-seq using bulk input and 50,000 cells were plotted for comparison.

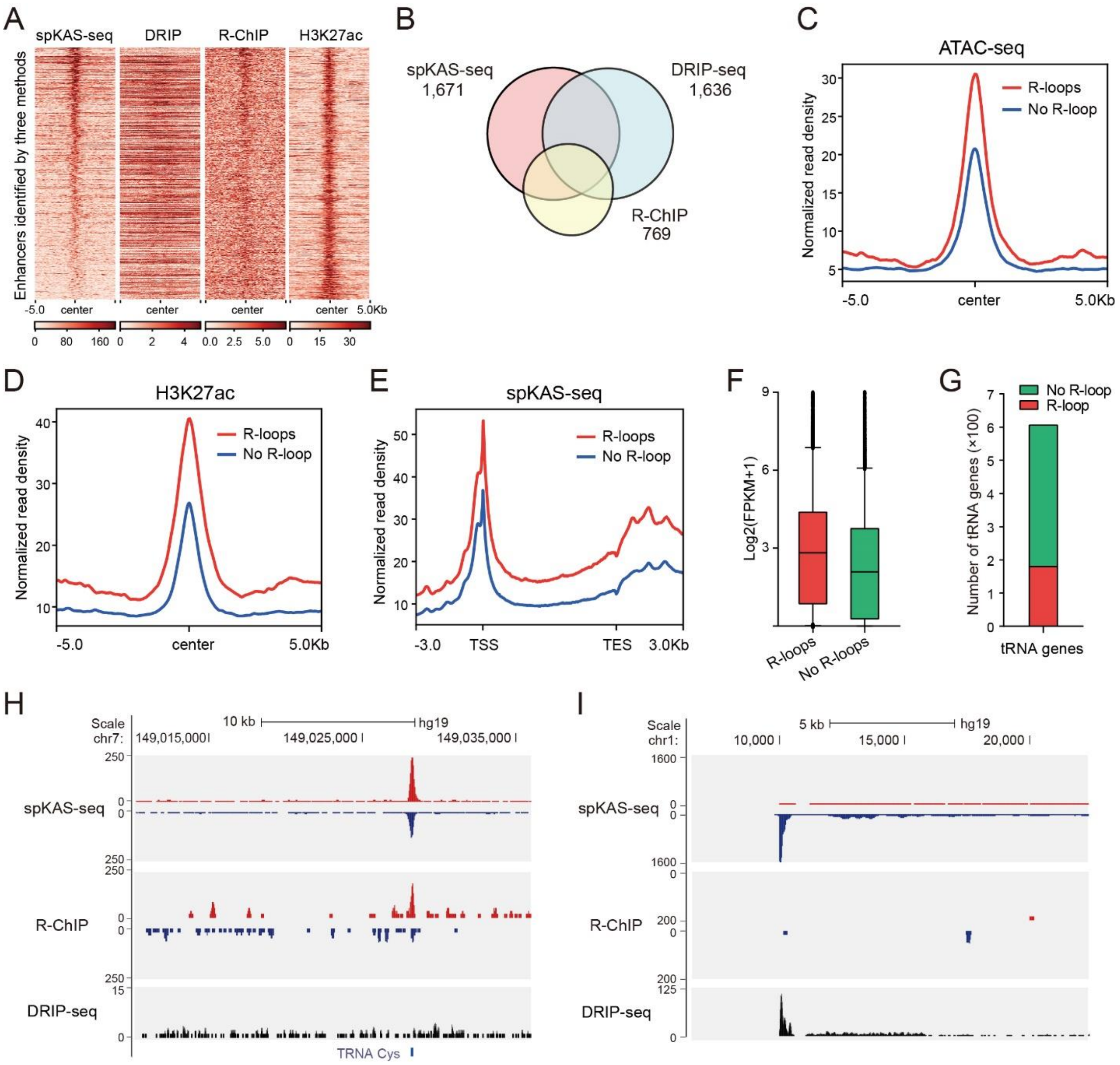

Figure S6. spKAS-seq detects R-loops on intergenic regions. (**A**) Heatmaps showing R-loops identified by spKAS-seq, DRIP-seq, and R-ChIP on enhancers in HEK293T cells. H3K27ac signals were shown as a reference. (**B**) The overlap between enhancer R-loops detected by spKAS-seq, DRIP-seq, and R-ChIP. (**C**)-(**D**) ATAC-seq (C) and H3K27ac (D) levels on active enhancers with or without R-loop signals in HEK293T cells. (**E**) Merged spKAS-seq signals on genes regulated by enhancers with or without R-loop signals in HEK293T cells. (**F**) A box plot showing the mRNA levels of genes regulated by enhancers with or without R-loop signals in HEK293T cells. The 5th to 95th percentile of data points were plotted, with the centerline depicting the median, and the box limits showing the upper and lower quartiles. (**G**) The amount of annotated tRNA genes that possess R-loop signals in HEK293T cells. (**H**) A representative snapshot showing spKAS-seq, R-ChIP, and DRIP-seq signals on a tRNA locus. (**I**) A representative snapshot showing spKAS-seq, R-ChIP, and DRIP-seq signals on a telomere-proximal region.

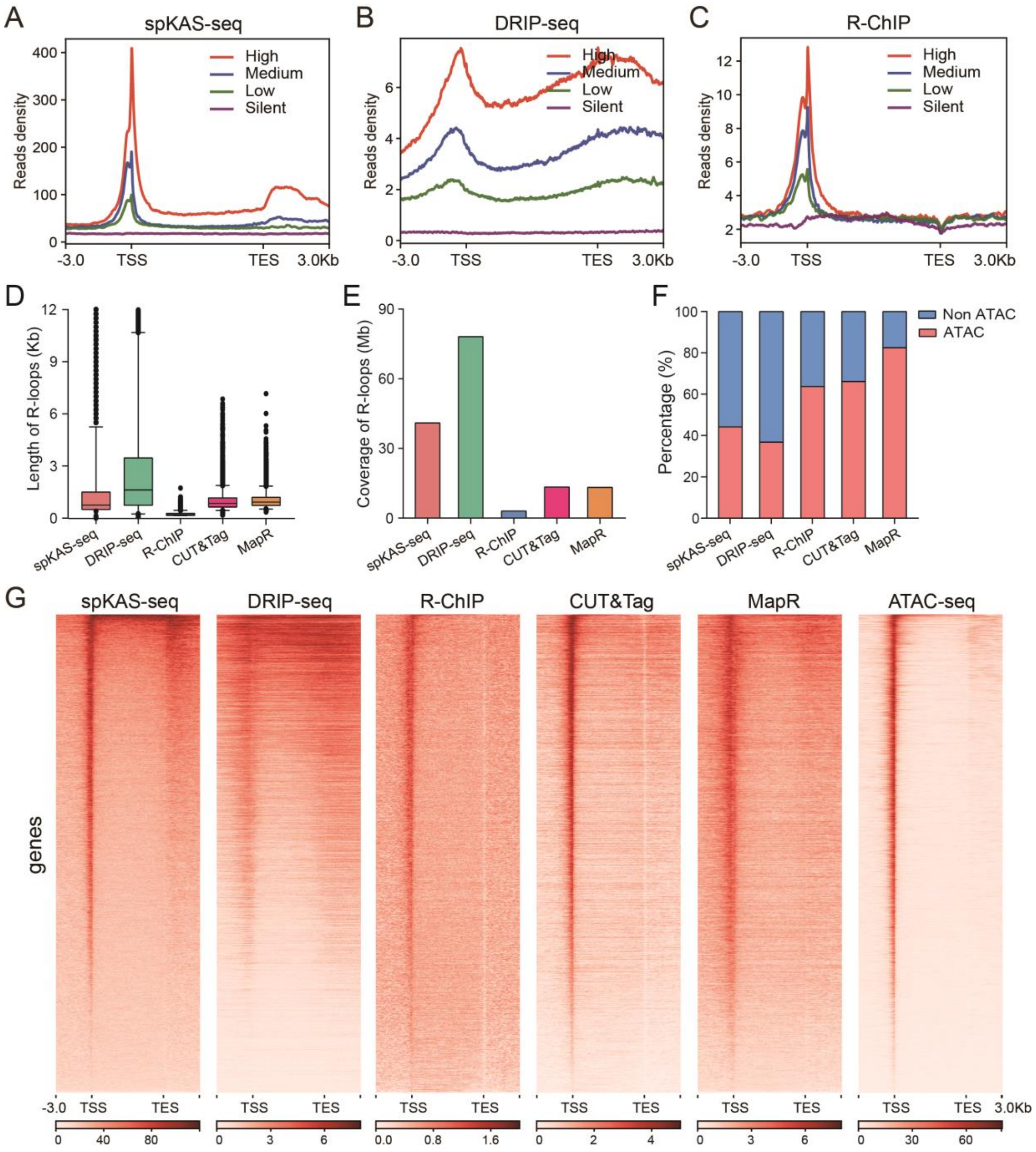

Figure S7. The comparison between different R-loop detection methods. (**A**)-(**C**) spKAS-seq, DRIP-seq, and R-ChIP profiles for R-loops at gene-coding regions. Genes were sorted into high, medium, low, and silent genes according to their mRNA levels and were depicted in red, blue, green, and purple, respectively. (**D**) The distribution of R-loop length revealed by spKAS-seq, DRIP-seq, R-ChIP, HBD CUT&Tag, and MapR. The 5th to 95th percentile of data points were plotted, with the centerline depicting the median, and the box limits showing the upper and lower quartiles. (**E**) The genomic coverage of R-loops identified by spKAS-seq, DRIP-seq, R-ChIP, HBD CUT&Tag, and MapR. (**F**) The portions of R-loops that overlap with ATAC-seq peaks for each denoted R-loop mapping method. (**G**) Heatmaps showing spKAS-seq, DRIP-seq, R-ChIP, HBD CUT&Tag, MapR, and ATAC-seq signals at gene-coding regions.

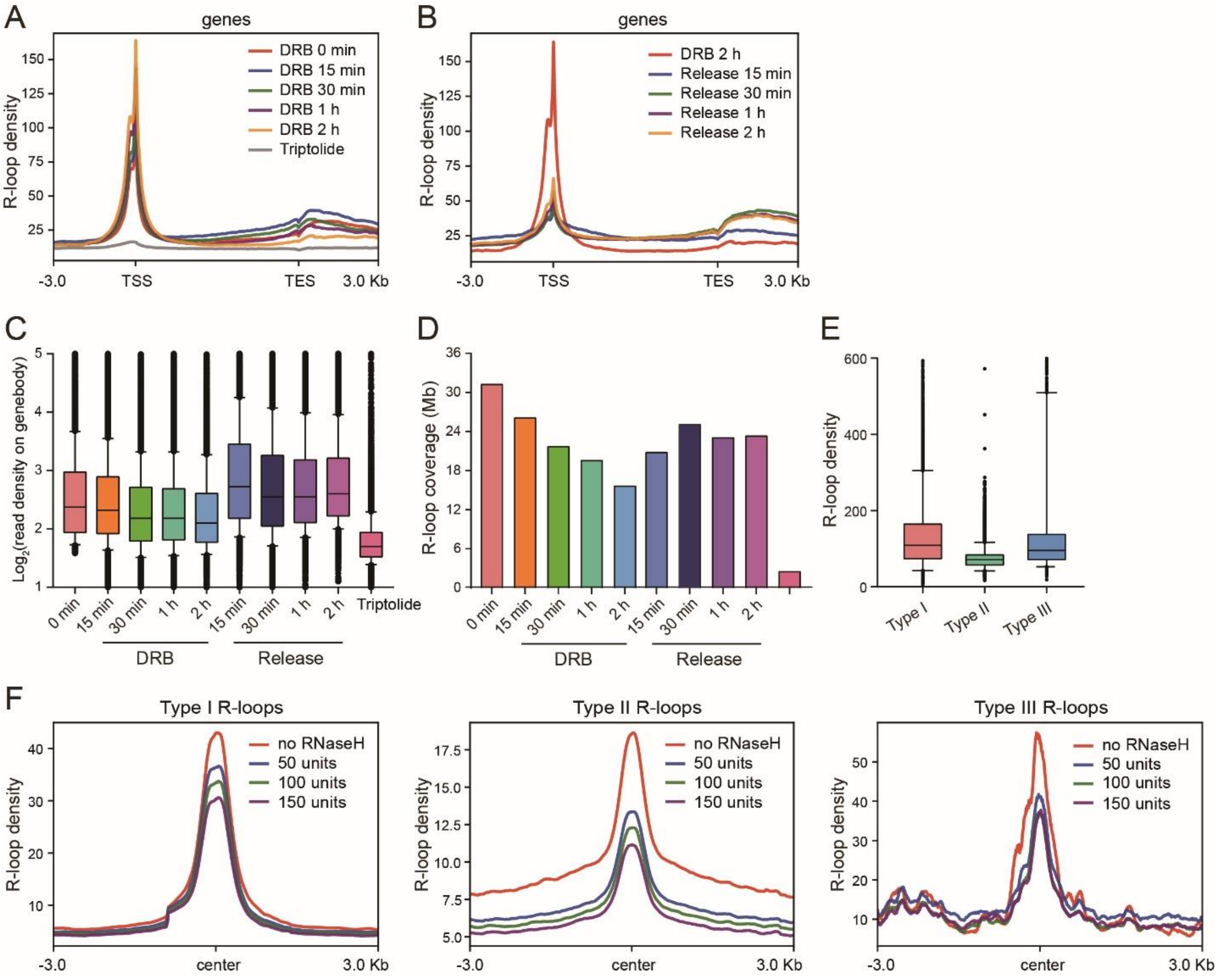

Figure S8. spKAS-seq reveals the response of R-loops to transcription perturbations. (**A**) R-loop density on gene-coding regions in HEK293T cells after DRB and triptolide treatment for denoted periods. (**B**) R-loop density on gene-coding regions in HEK293T cells at denoted time points after DRB was washed away. Cells were treated with DRB for 2 h before DRB was removed. (**C**) spKAS-seq reads density in gene bodies at denoted time points after DRB treatment, DRB removal, or triptolide treatment. (**D**) R-loop genomic coverage at denoted time points after DRB treatment, DRB removal, or triptolide treatment. (**E**) The density of three types of R-loops identified by spKAS-seq. (**F**) Metagene analysis for the levels of three types of R-loops in HEK293T cells treated by different dosages of RNase H. For box plots in (C) and (E), the 5th to 95th percentile of data points were plotted, with the centerline depicting the median, and the box limits showing the upper and lower quartiles.

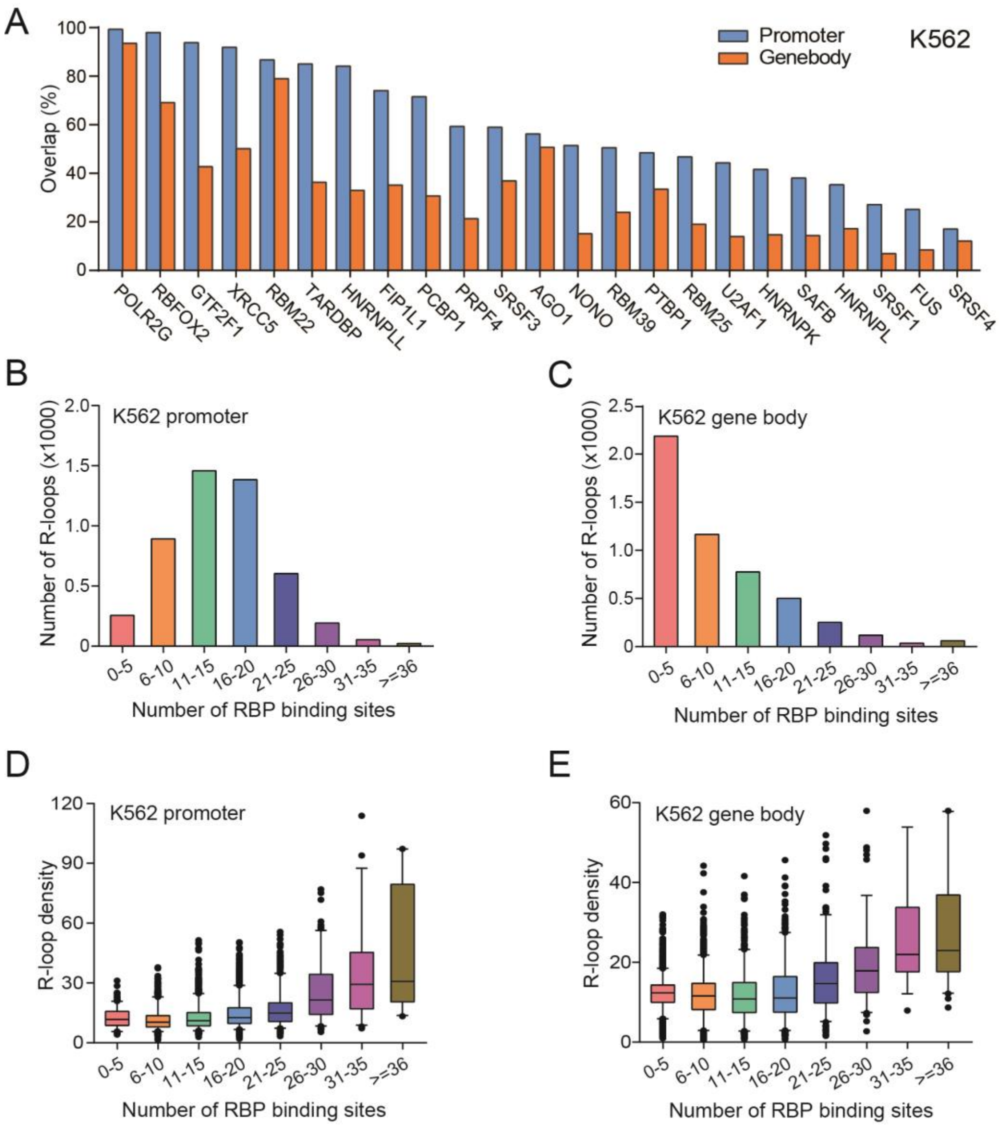

Figure S9. The association between R-loops and RBP-binding on the chromatin in K562 cells. (**A**) The percentages of R-loops that overlap with ChIP-seq peaks of each denoted RBP at promoters and gene bodies in K562 cells. (**B**)-(**C**) The numbers of promoter (B) and gene body (C) R-loops that overlap with different numbers of RBP ChIP-seq peaks in K562 cells. (**D**)-(**E**) The relationship between the number of RBP ChIP-seq peaks and the strength of R-loops in K562 promoters (D) and gene bodies (E). The 5th to 95th percentile of data points were plotted, with the centerline depicting the median, and the box limits showing the upper and lower quartiles.

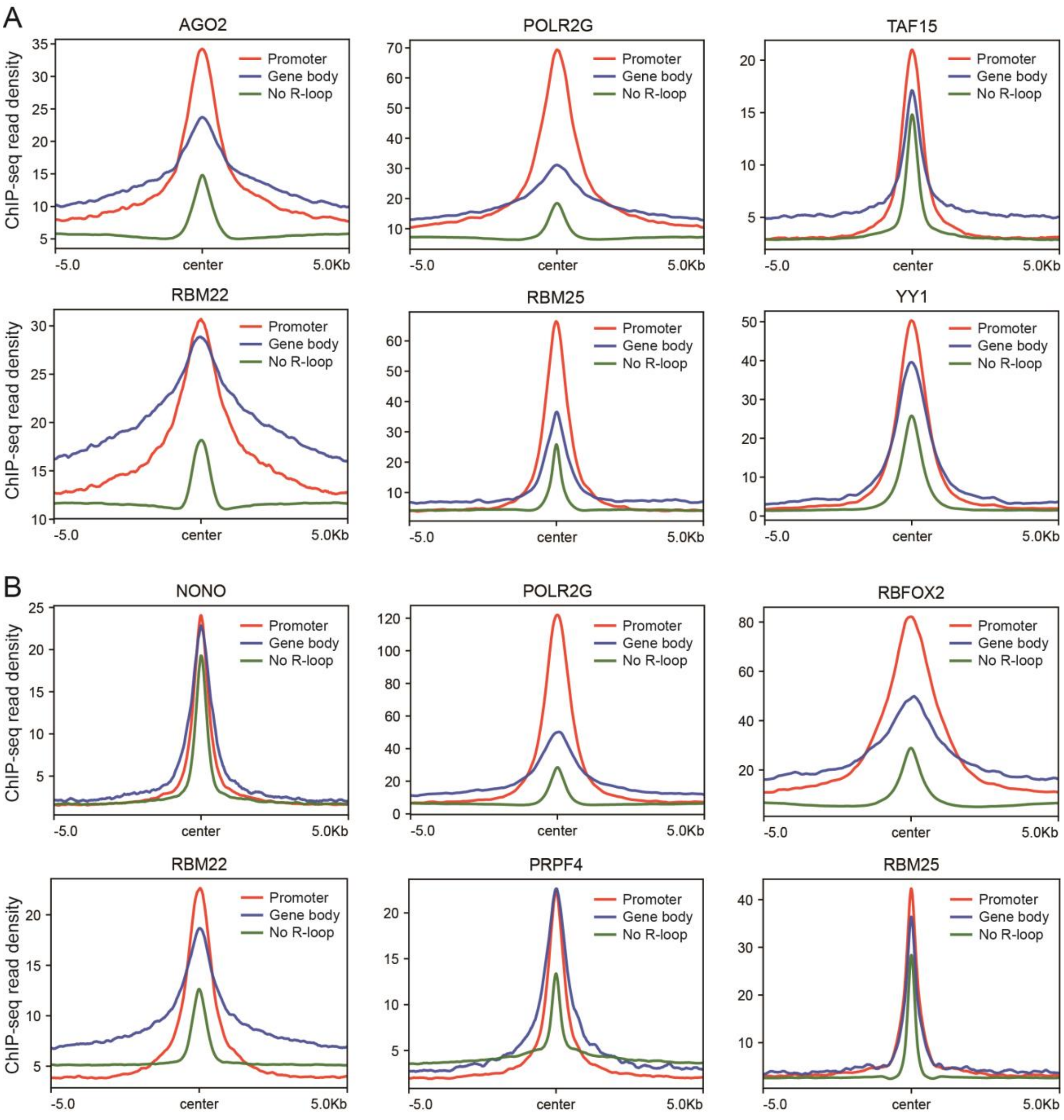

Figure S10. The effect of R-loops on RBP binding. RBP ChIP-seq reads density on ChIP-seq peaks that overlap with promoter R-loops (red), gene body R-loops (blue), and regions with no R-loops (green) in HepG2 (**A**) and K562 cells (**B**).

Data S1. (separate file) High-throughput sequencing data generated in this study.

Data S2. (separate file) Public data used in this study.
